# Supplementary material for: Algorithmic assessment of shoulder function using smartphone video capture and machine learning
Source: Sci Rep. 2023 Nov 15;13:19986. doi: 10.1038/s41598-023-46966-4 (PMC10652003; doi:10.1038/s41598-023-46966-4)
Supplement: Supplementary file 1 — Supplementary Information 1. [file 41598_2023_46966_MOESM1_ESM.pdf]

Supporting Information: A Tool for Low-Cost, Quantitative Assessment of Shoulder Function Using Machine Learning

David M. Darevsky[1,2,3,4,5,6*], Daniel A. Hu[3,5*], Francisco A. Gomez[3,5], Michael R. Davies[3,5], Xuhui Liu[3,5], Brian T. Feeley[3,5]

[1]Bioengineering Graduate Program, University of California San Francisco and University of California Berkeley, San Francisco, CA and Berkeley, CA.

[2]Medical Scientist Training Program, University of California San Francisco, San Francisco, CA.

[3]University of California, San Francisco, Department of Orthopaedic Surgery

[4]Department of Neurology, University of California San Francisco, San Francisco, CA.

[5]San Francisco Veterans Affairs Health Care System

[6]Neurology and Rehabilitation Service, San Francisco Veterans Affairs Medical Center, San Francisco, CA, USA.

*These authors contributed equally

Correspondence to: Brian Feeley, brian.feeley@ucsf.edu

Contents:
Supplemental Tables 1-3
Supplemental Figures 1-7

Supplementary Table 1: Cost of materials for conducting both rodent and human string pulling experiments. All values rounded to the nearest dollar.

Item	Unit Cost	Source	Quantity	Sub-total
Plexiglass behavior box (5 x 6 x 9 in)	$40	Casesforcollectibles.com	1	$40
Red string for mouse experiments (1.5mm ⌀)	$5	Amazon.com	1	$5
Manfrotto Compact Action Aluminum Tripod	$70	Amazon.com	1	$70
Manfrotto Smartphone Clamp	$10	Amazon.com	1	$70
Smartphone for video recording	Variable	N/A	1	
3D printed string holder	~$5 per print	N/A	1	$5
Shoulder pulley	$13	Amazon.com	1	$13
Red rope for human experiments (6 mm ⌀)	$15	Amazon.com	1	$15
NVIDIA 2080 Ti GPU	$1000	BestBuy.com	1	$1000
Desktop computer	Variable	N/A	1	
				**Total = $1,218**

Supplementary Table 2, Statistics

Figure 2					
**Panel**	**Biological replicates**	**Comparison**	**Test**	***P* value**	***P* value summary**
**e**	n=6 with no repair, n=6 with immediate repair	**Right hand repair vs. no repair**	**Two-sided Kolmogorov-Smirnoff test**	0.2001	ns
**e**	n=6 with no repair, n=6 with immediate repair	**Left hand repair vs. no repair**	**Two-sided Kolmogorov-Smirnoff test**	0.2069	ns

Figure 3					
**Panel**	**Biological replicates**	**Comparison**	**Test**	***P* value**	***P* value summary**
**a**	12	**FWHM: effect of arm (injured vs. uninjured)**	**two-way ANOVA**	0.833	ns
**a**	12	**FWHM: effect of time**	**two-way ANOVA**	0.740	ns
**a**	12	**FWHM: effects of arm (injured vs. uninjured) and time**	**two-way ANOVA**	<0.001	***
		Follow-up: Injured vs. uninjured arm at Week 1	Tukey post-hoc test (corrected)	<0.001	***
		Follow-up: Injured vs. uninjured arm at Week 2	Tukey post-hoc test (corrected)	<0.001	***
		Follow-up: Injured vs. uninjured arm at Week 3	Tukey post-hoc test (corrected)	<0.001	***

		Follow-up: Injured vs. uninjured arm at Week 4	Tukey post-hoc test (corrected)	<0.001	***
		Follow-up: Injured vs. uninjured arm at Baseline	Tukey post-hoc test (corrected)	1.0	ns
**b**	12	**Central tendency of the mean velocity: effects of time**	**two-way ANOVA**	0.733	ns
**b**	12	**Central tendency of the mean velocity: effect of arm (injured vs. uninjured)**	**two-way ANOVA**	0.659	ns
**b**	12	**Central tendency of the mean velocity: effects of arm (injured vs. uninjured) and time**	**two-way ANOVA**	0.505	ns
**b**	12	**Variance of velocity distribution: effect of injured vs. uninjured arms at Baseline**	**Levene's test for equality of variance**	0.002	**
**b**	12	**Variance of velocity distribution: effect of injured vs. uninjured**	**Levene's test for equality of variance**	<0.001	***

		**arms at Week 1**			
**b**	12	**Variance of velocity distribution: effect of injured vs. uninjured arms at Week 2**	**Levene's test for equality of variance**	<0.001	***
**b**	12	**Variance of velocity distribution: effect of injured vs. uninjured arms at Week 3**	**Levene's test for equality of variance**	<0.001	***
**b**	12	**Variance of velocity distribution: effect of injured vs. uninjured arms at Week 4**	**Levene's test for equality of variance**	<0.001	***
**c**	12	**Mean acceleration: effect of time**	**two-way ANOVA**	<0.001	***
**c**	12	**Mean acceleration: effect of arm (injured vs. uninjured)**	**two-way ANOVA**	<0.001	***
**c**	12	**Mean acceleration: effects of arm (injured vs. uninjured) and time**	**two-way ANOVA**	<0.001	***
		Follow-up: Injured vs. uninjured arm at Baseline	Tukey post-hoc test (corrected)	0.001	***

		Follow-up: Injured vs. uninjured arm at Week 1	Tukey post-hoc test (corrected)	<0.001	***
		Follow-up: Injured vs. uninjured arm at Week 2	Tukey post-hoc test (corrected)	<0.001	***
		Follow-up: Injured vs. uninjured arm at Week 3	Tukey post-hoc test (corrected)	<0.001	***
		Follow-up: Injured vs. uninjured arm at Week 4	Tukey post-hoc test (corrected)	<0.001	***
**c**	12	**Variance of acceleration distribution: effect of injured vs. uninjured arms at Baseline**	**Levene's test for equality of variance**	<0.001	***
**c**	12	**Variance of acceleration distribution: effect of injured vs. uninjured arms at Week 1**	**Levene's test for equality of variance**	<0.001	***
**c**	12	**Variance of acceleration distribution: effect of injured vs. uninjured arms at Week 2**	**Levene's test for equality of variance**	<0.001	***

**c**	12	**Variance of acceleration distribution: effect of injured vs. uninjured arms at Week 3**	**Levene's test for equality of variance**	<0.001	***
**c**	12	**Variance of acceleration distribution: effect of injured vs. uninjured arms at Week 4**	**Levene's test for equality of variance**	<0.001	***

Figure 4					
**Panel**	**Biological replicates**	**Comparison**	**Test**	***P* value**	***P* value summary**
**a**	12	**PC1 variance: effect of time**	**One-way ANOVA**	<0.001	***
		Follow-up: Baseline vs. Week 1	Tukey post-hoc test (corrected)	0.006	**
		Follow-up: Baseline vs. Week 2	Tukey post-hoc test (corrected)	0.015	*
		Follow-up: Baseline vs. Week 3	Tukey post-hoc test (corrected)	0.006	**
		Follow-up: Baseline vs. Week 4	Tukey post-hoc test (corrected)	0.093	ns
**a**	12	**PC2 variance: effect of time**	**One-way ANOVA**	<0.001	***
		Follow-up: Baseline vs. Week 1	Tukey post-hoc test (corrected)	0.019	*
		Follow-up: Baseline vs. Week 2	Tukey post-hoc test (corrected)	0.546	ns

		Follow-up: Baseline vs. Week 3	Tukey post-hoc test (corrected)	0.001	***
		Follow-up: Baseline vs. Week 4	Tukey post-hoc test (corrected)	0.347	ns
**b**	12	**Absolute value of eigenvector weights: effect of arm (injured vs. uninjured)**	**Two-way ANOVA**	0.554	ns
**b**	12	**Absolute value of eigenvector weights: effect of time**	**Two-way ANOVA**	0.455	ns
**b**	12	**Absolute value of eigenvector weights: effects of arm (injured vs. uninjured) and time**	**Two-way ANOVA**	0.045	*
		Follow-up: Injured vs. uninjured arm at Week 1	Tukey post-hoc test (corrected)	0.006	**
		Follow-up: Injured vs. uninjured arm at Week 2	Tukey post-hoc test (corrected)	0.081	.
		Follow-up: Injured vs. uninjured arm at Week 3	Tukey post-hoc test (corrected)	0.004	**
**d**	12	**Bispectral coherence values: effect of time**	**One-way ANOVA**	0.079	.

Figure 5					
**Panel**	**Biological replicates**	**Comparison**	**Test**	***P* value**	***P* value summary**
**a**	12	**Movement amplitude: effect of time**	**Three-way ANOVA**	<0.001	***
**a**	12	**Movement amplitude: effect of arm (injured vs. uninjured)**	**Three-way ANOVA**	0.063	.
**a**	12	**Movement amplitude: effects of time and arm (injured vs. uninjured)**	**Three-way ANOVA**	<0.001	***
**a**	12	**Movement amplitude: effect of movement epoch**	**Three-way ANOVA**	0.445	ns
		Follow-up: Baseline vs. Week 1 for injured arm	Tukey post-hoc test (corrected)	<0.001	***
		Follow-up: Baseline vs. Week 2 for injured arm	Tukey post-hoc test (corrected)	0.0639	.
		Follow-up: Baseline vs. Week 3 for injured arm	Tukey post-hoc test (corrected)	0.0763	.
		Follow-up: Baseline vs. Week 4 for injured arm	Tukey post-hoc test (corrected)	0.9993	ns
		Follow-up: Baseline vs. Week 1 for uninjured arm	Tukey post-hoc test (corrected)	<0.001	***

		Follow-up: Baseline vs. Week 2 for uninjured arm	Tukey post-hoc test (corrected)	0.9984	ns
		Follow-up: Baseline vs. Week 3 for uninjured arm	Tukey post-hoc test (corrected)	<0.001	***
		Follow-up: Baseline vs. Week 4 for uninjured arm	Tukey post-hoc test (corrected)	<0.001	***
**b**	12	**Movement amplitude: effect of time**	**Three-way ANOVA**	<0.001	***
**b**	12	**Movement amplitude: effect of arm (injured vs. uninjured)**	**Three-way ANOVA**	0.680	ns
**b**	12	**Movement amplitude: effects of time and arm (injured vs. uninjured)**	**Three-way ANOVA**	0.058	.
**b**	12	**Movement amplitude: effect of movement epoch**	**Three-way ANOVA**	<0.001	***
**b**	12	**Bispectral coherence values: effect of time**	**One-way ANOVA**	0.079	.
		Follow-up: Baseline vs. Week 1 for time	Tukey post-hoc test (corrected)	<0.001	***
		Follow-up: Baseline vs.	Tukey post-hoc test (corrected)	<0.001	***

		Week 2 for time			
		Follow-up: Baseline vs. Week 3 for time	Tukey post-hoc test (corrected)	<0.001	***
		Follow-up: Baseline vs. Week 4 for time	Tukey post-hoc test (corrected)	<0.001	***

Figure 6					
**Panel**	**Biological replicates**	**Comparison**	**Test**	***P* value**	***P* value summary**
**c**	n=6 controls (uninjured shoulder), n=6 patients (injured shoulder)	**FWHM: control vs. injured shoulders**	**Two-sided Kolmogorov-Smirnoff test**	<0.001	***
**d, left**	n=6 control uninjured, n=6 injured shoulder, n=6 contralateral uninjured	**Velocity: control vs. injured vs. contralateral uninjured**	**One-way Kruskal-Wallis test**	<0.001	***
		Follow-up: Control vs. Injured	Mann-Whitney U test post-hoc (corrected)	<0.001	***
		Follow-up: Control vs. Contralateral uninjured	Mann-Whitney U test post-hoc (corrected)	<0.001	***
		Follow-up: Control vs. Contralateral uninjured	Mann-Whitney U test post-hoc (corrected)	0.021	*
**d, right**	n=6 control uninjured, n=6 injured shoulder, n=6 contralateral uninjured	**Acceleration: control vs. injured vs. contralateral uninjured**	**One-way Kruskal-Wallis test**	<0.001	***

		Follow-up: Control vs. Injured	Mann-Whitney U test post-hoc (corrected)	0.001	***
		Follow-up: Control vs. Contralateral uninjured	Mann-Whitney U test post-hoc (corrected)	<0.001	***
		Follow-up: Control vs. Contralateral uninjured	Mann-Whitney U test post-hoc (corrected)	1	ns
**e**	n=6 control uninjured, n=6 injured shoulder, n=6 contralateral uninjured	**Y-axis movements: control vs. injured vs. contralateral uninjured**	**One-way ANOVA**	0.086	.
**f**	n=6 control uninjured, n=6 injured shoulder, n=6 contralateral uninjured	**Movement amplitude: effect of movement epoch (reach vs. pull)**	**Two-way ANOVA**	0.672	ns
**f**	n=6 control uninjured, n=6 injured shoulder, n=6 contralateral uninjured	**Movement amplitude: effect of arm (control vs. injured vs. contralateral uninjured)**	**Two-way ANOVA**	0.004	**
**f**	n=6 control uninjured, n=6 injured shoulder, n=6 contralateral uninjured	**Movement amplitude: effects of movement epoch (reach vs. pull) and arm (control vs. injured vs. contralateral uninjured)**	**Two-way ANOVA**	0.067	.
		Follow-up: Control vs. Injured	Tukey post-hoc test (corrected)	<0.001	***

		Follow-up: Injured vs. Contralateral uninjured	Tukey post-hoc test (corrected)	<0.001	***
		Follow-up: Control vs. Contralateral uninjured	Tukey post-hoc test (corrected)	<0.001	***
**g**	n=6 control uninjured, n=6 injured shoulder, n=6 contralateral uninjured	**Mean time: effect of movement epoch (reach vs. pull)**	**Two-way ANOVA**	0.011	*
**g**	n=6 control uninjured, n=6 injured shoulder, n=6 contralateral uninjured	**Mean time: effect of arm (control vs. injured vs. contralateral uninjured)**	**Two-way ANOVA**	<0.001	***
**g**	n=6 control uninjured, n=6 injured shoulder, n=6 contralateral uninjured	**Mean time: effects of movement epoch (reach vs. pull) and arm (control vs. injured vs. contralateral uninjured)**	**Two-way ANOVA**	0.004	**
		Follow-up: Control vs. Injured for pull time	Tukey post-hoc test (corrected)	<0.001	***
		Follow-up: Control vs. Contralateral uninjured for pull time	Tukey post-hoc test (corrected)	<0.001	***
		Follow-up: Control reach time vs. Injured pull time	Tukey post-hoc test (corrected)	<0.001	***

		Follow-up: Injured pull time vs. Contralateral uninjured reach time	Tukey post-hoc test (corrected)	<0.001	***
		Follow-up: Control reach time vs. Contralateral uninjured pull time	Tukey post-hoc test (corrected)	<0.001	***
		Follow-up: Injured reach time vs. Contralateral uninjured pull time	Tukey post-hoc test (corrected)	<0.001	***
		Follow-up: Contralateral uninjured reach time vs. Contralateral uninjured pull time	Tukey post-hoc test (corrected)	<0.001	***
**h**	n=6 control uninjured, n=6 injured shoulder, n=6 contralateral uninjured	**Dynamic range ratio: effect of arm (control vs. injured vs. contralateral uninjured)**	**One-way ANOVA**	0.008	**
		Follow-up: Control vs. Injured extremities	Tukey post-hoc test (corrected)	0.006	**

Supplementary Figure 5					
**Panel**	**Biological replicates**	**Comparison**	**Test**	***P* value**	***P* value summary**
	n=6 rotator cuffs, n=6 controls	**Variability explained by each PC: effect of group**	**Two-way ANOVA**	0.237	ns

		**(rotator cuff vs. control)**			
	n=6 rotator cuffs, n=6 controls	**Variability explained by each PC: effect of PC number**	**Two-way ANOVA**	<0.001	***
	n=6 rotator cuffs, n=6 controls	**Variability explained by each PC: effect of PC number and group (rotator cuff vs. control)**	**Two-way ANOVA**	0.171	ns

Supplementary Table 3. Patient demographic data for patients with diagnosed rotator cuff tears.

Subject ID	Diagnosis	Laterality	Age	Duration of Symptoms
**1**	Mild RC tear	right	73	>2 years
**2**	Recent mild RC tear	right	67	3 months
**3**	Recent mild RC tear, biceps tendon injury	left	45	1 month
**4**	Massive RC tear	left	57	1 year
**5**	Recent moderate RC tear	left	79	3 months
**6**	Moderate RC tear	left	63	>1 year

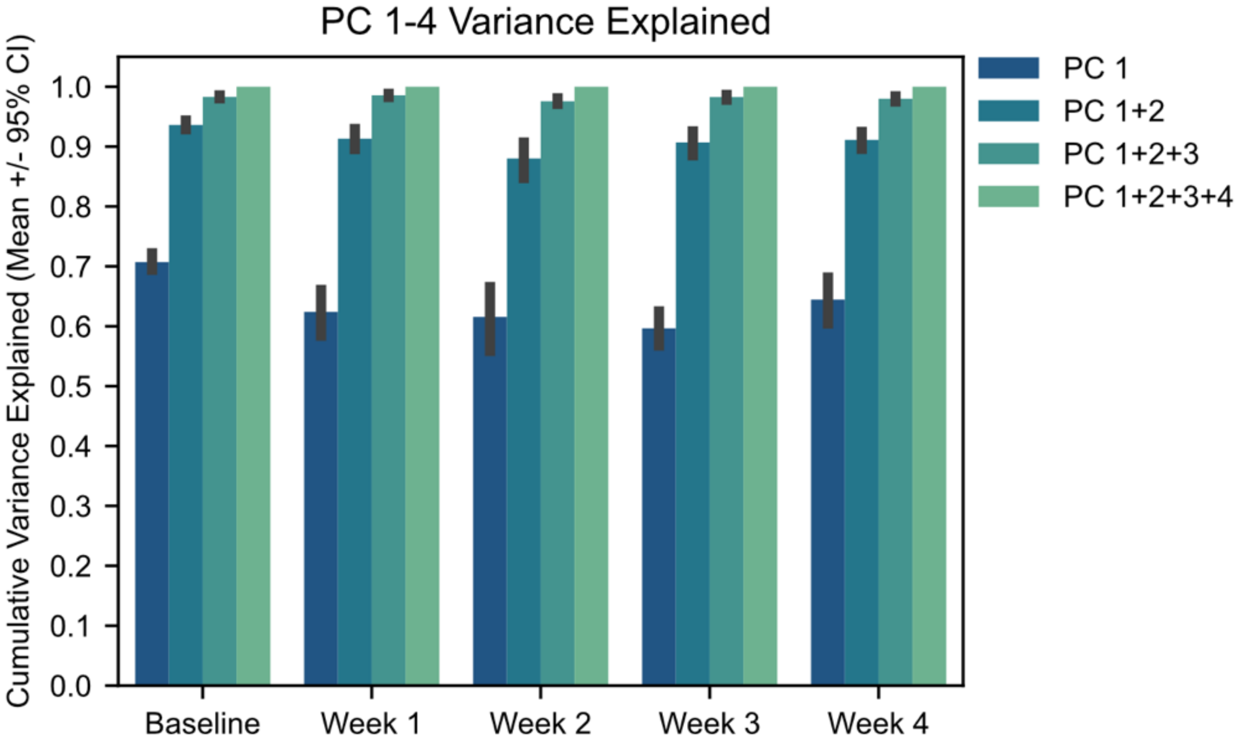

Supplementary Figure 1 – Cumulative variance explained by each principal component (PC) across all five experimental time points. n = 12 mice. Data shown as Mean ± SEM.

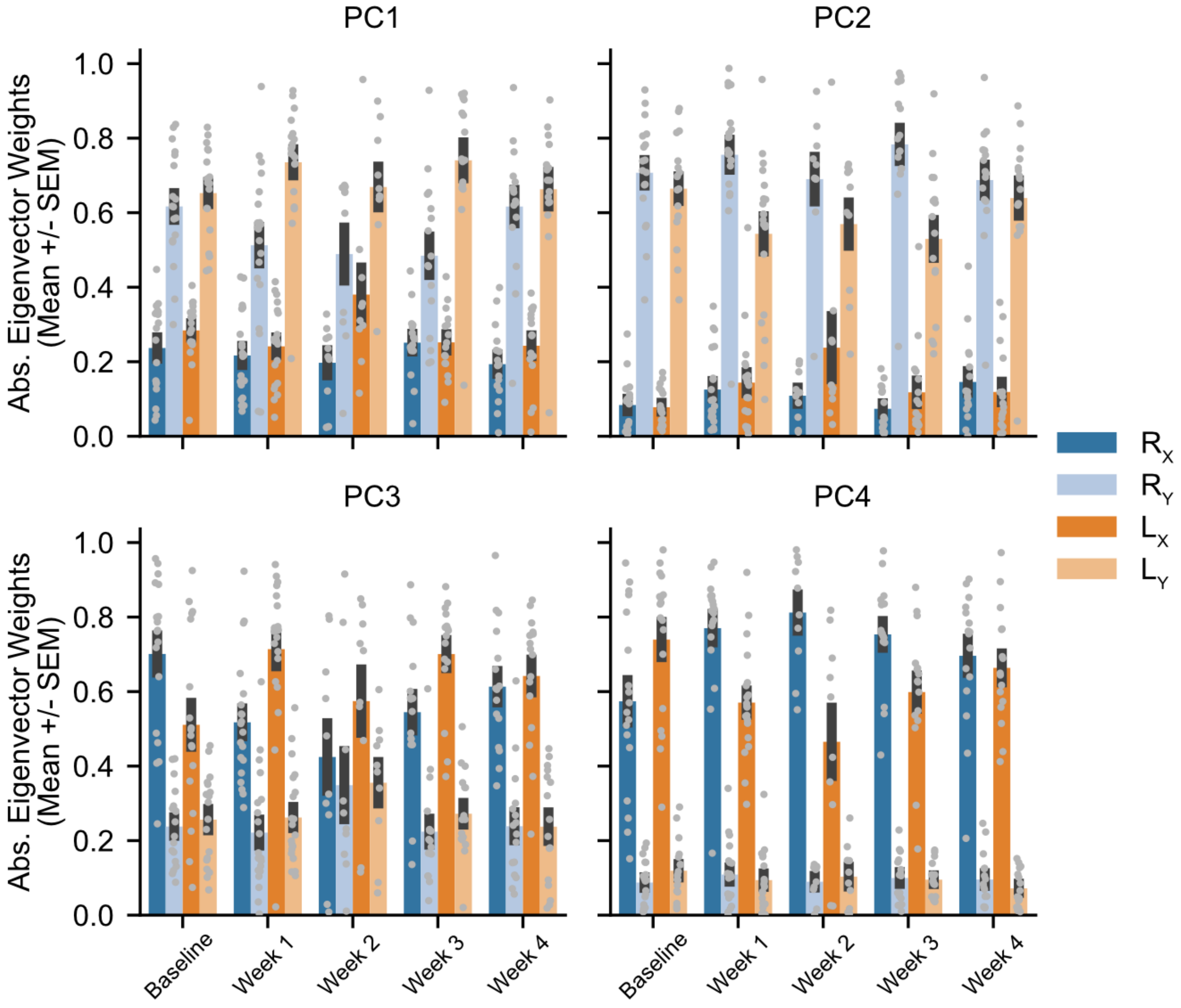

Supplementary Figure 2 – Absolute right singular vector weights shown for Principal Componenents (PCs) 1 through 4 across all recording time points. *Gray* dots represent individual singular vector weights from each animal's video recordings. Data shown as Mean ± SEM.

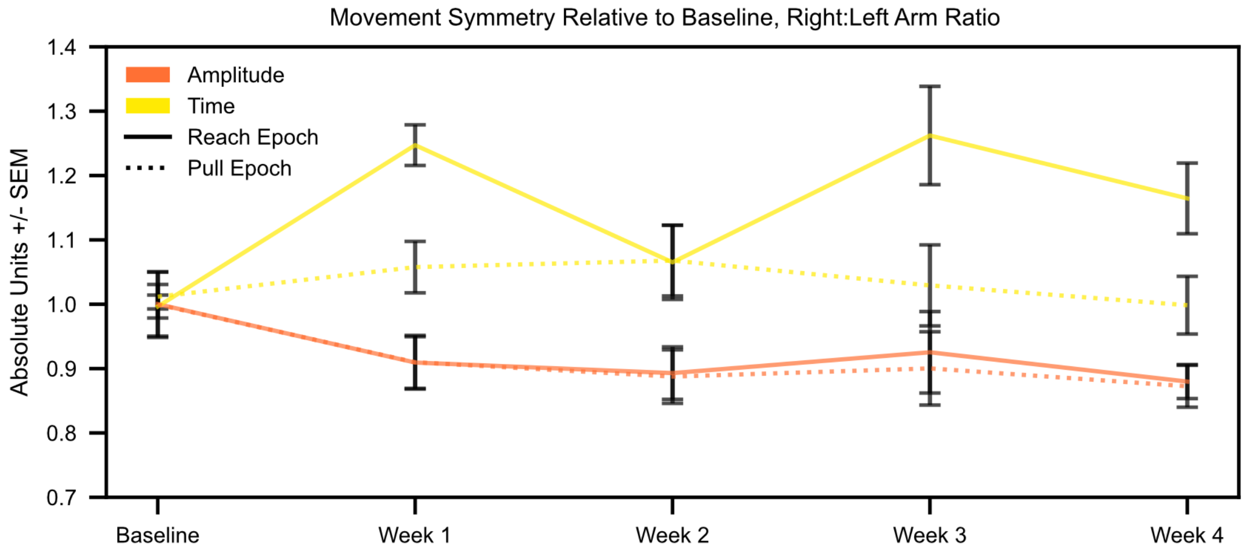

Supplementary Figure 3 – Symmetry measure for amplitude (orange lines) and time (yellow lines). Data shown as ratio of right:left hand values calculated for every video by dividing mean amplitude/time for all reach/pull epochs. Data shown as Mean ± SEM.

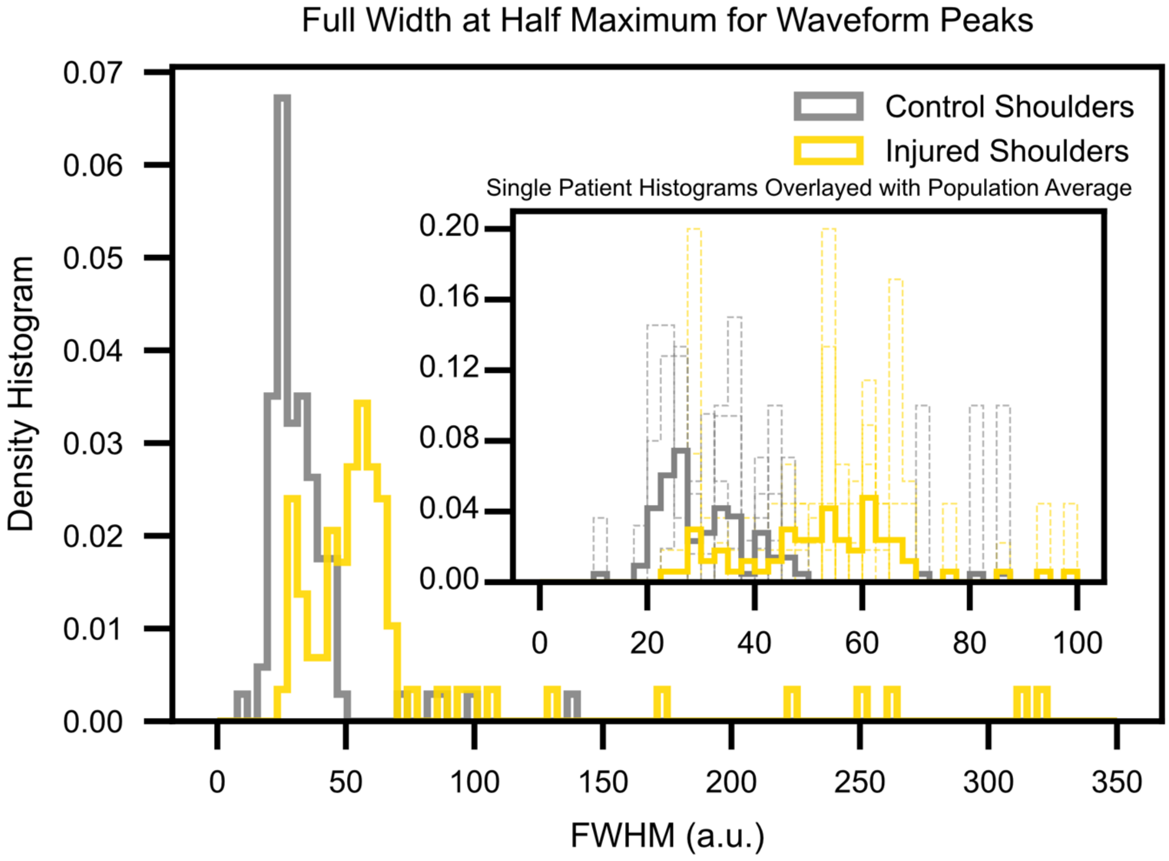

Supplementary Figure 4 – Same histogram plot of full-width at half maximum (FWHM) values as shown in Fig. 6c (control shoulders in *gray*, injured shoulders in *yellow*). FWHM values for single subjects are plotted as *dotted* lines within the inset plot.

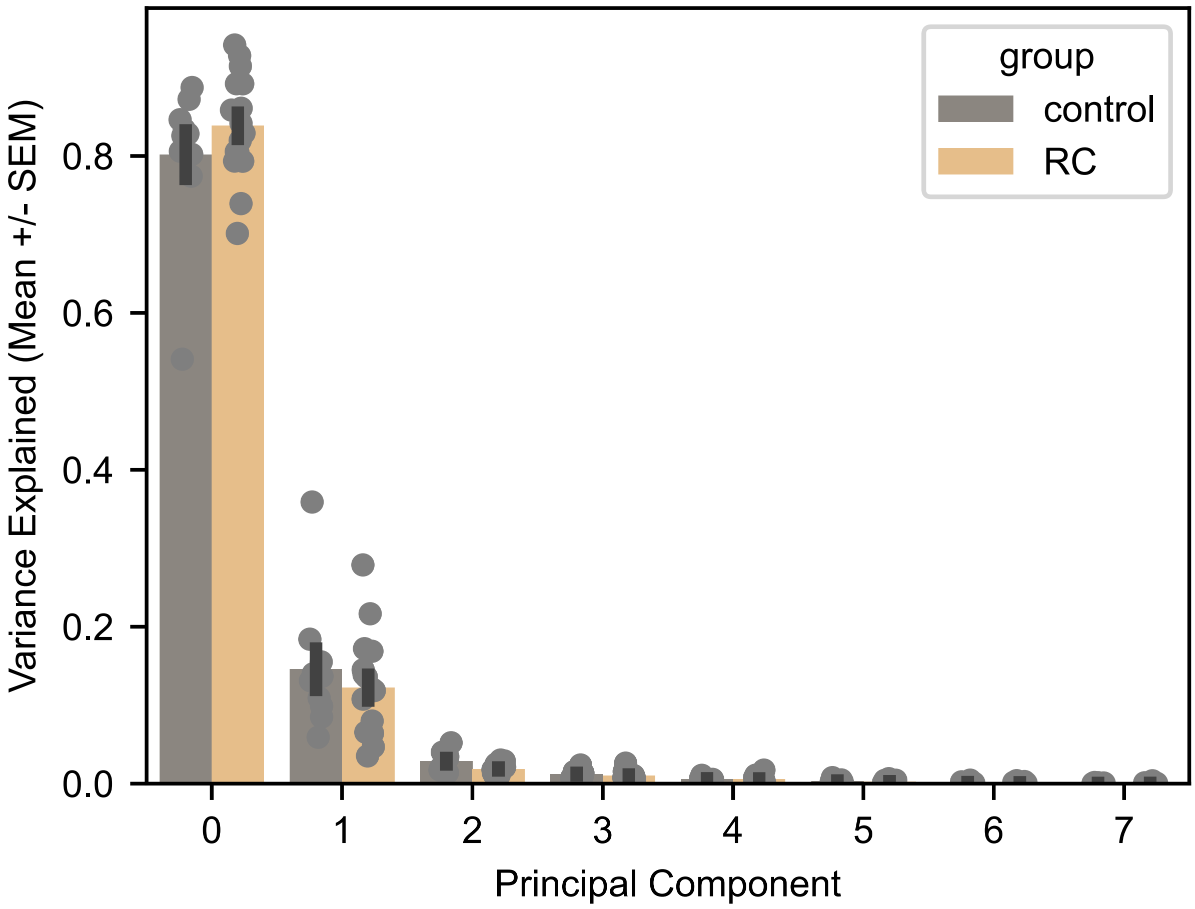

Supplementary Figure 5 – Plot of variance explained by each principal component across control shoulders (*gray* bars) and shoulders with known rotator cuff tear (RC, *tan* bars). Individual dots show values from each study participant. Data presented as Mean ± SEM.

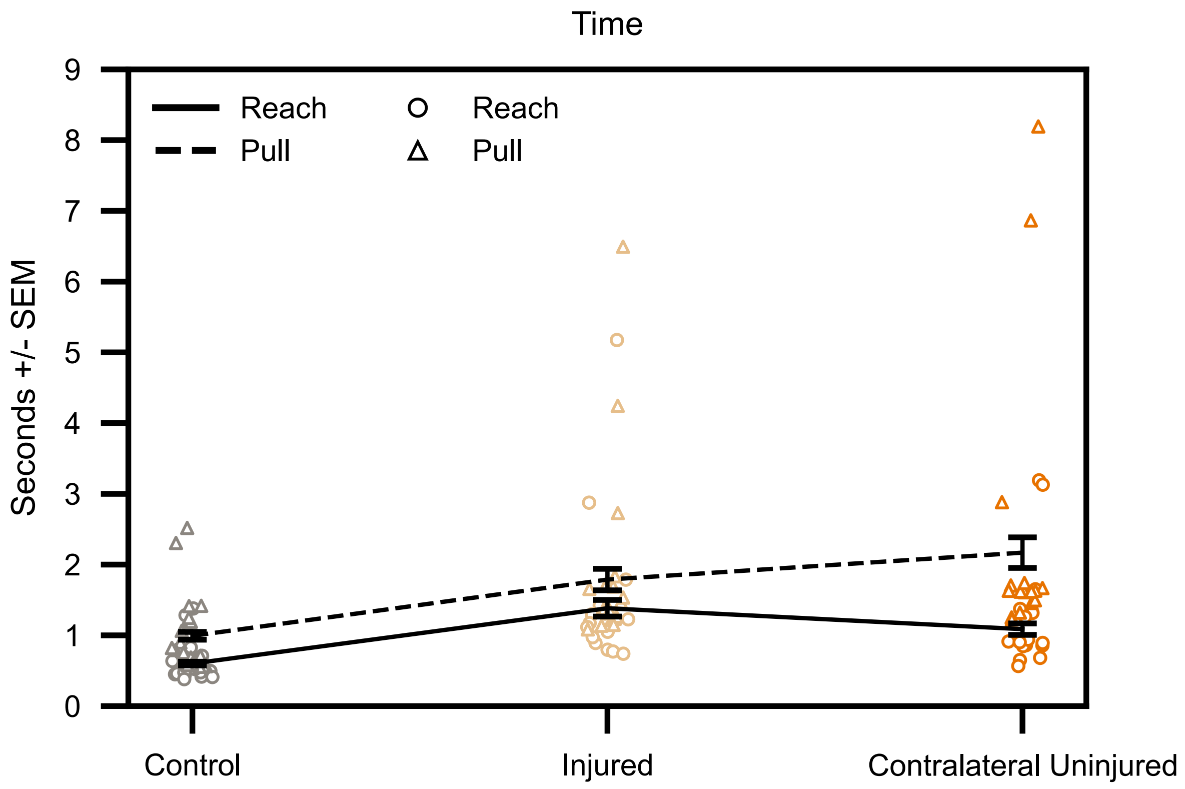

Supplementary Figure 6 - Plot of reach and pull times across control (*gray* dots), injured (*tan* dots), and contralateral uninjured (*orange* dots) shoulders. Same data as main Fig. 6g only with four outlier points shown. Time of reach and pull epochs shown by *solid* and *dashed* lines, respectively. Individual time values from every participant shown for reaches and pulls with *circles* and *triangles*, respectively

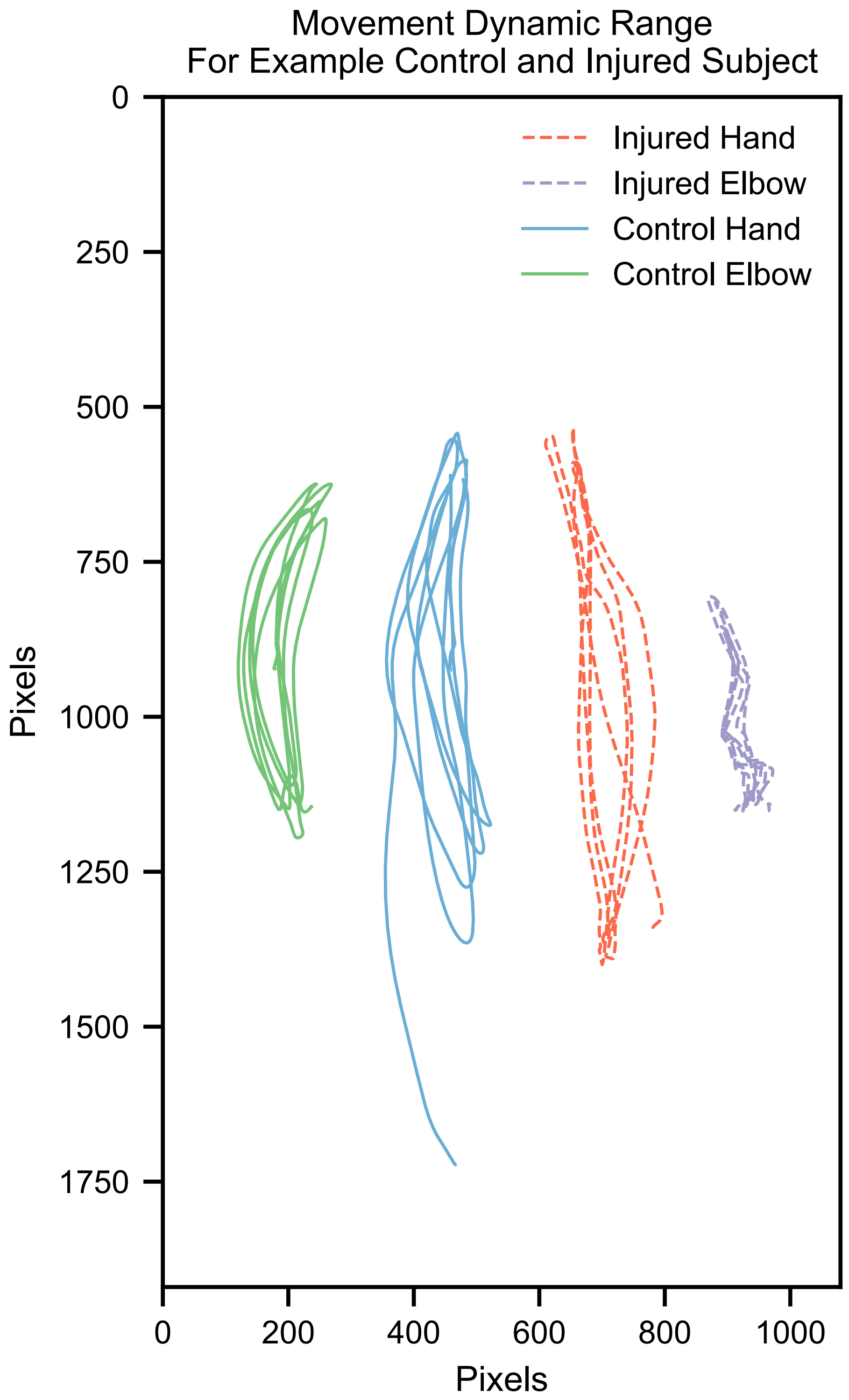

Supplementary Figure 7 - Representative traces for control subject (*solid* lines) and a subject with known rotator cuff tear (*dashed* lines). Note loss of elbow motion (*purple dashed* line) relative to hand motion (*red dashed* line) for the subject with a known rotator cuff tear
